# A systematic review and meta-analysis of the effects of Entertainment-Education interventions on persuasive health outcomes

**DOI:** 10.1038/s41598-025-11012-y

**Published:** 2025-07-31

**Authors:** Anastasia Doré, Felix G. Rebitschek, Karolina Morello, Elena Stierstädter, Kyra J. Thiemann, Thomas Kubiak

**Affiliations:** 1 Health Psychology, Institute of Psychology, Johannes Gutenberg University Mainz, Mainz, Germany; 2 Harding Center for Risk Literacy, Faculty of Health Sciences Brandenburg, University of Potsdam, Potsdam, Germany; 3 Max Planck Institute for Human Development, Berlin, Germany; 4 Leibniz Institute for Resilience Research (LIR), Leibniz Association, Mainz, Germany

**Keywords:** Health communication, Public health psychology, Persuasion, Narratives, Entertainment-Education, Systematic review, Psychology, Health care

## Abstract

Entertainment-Education (EE) refers to the integration of entertaining and educational components within popular media, with the objective of positively impacting social and health behaviors. This study aims to synthesize the effects of EE for health communication. In particular, the effects of EE on health knowledge, intentions, attitudes, behavior, and self-efficacy were analyzed. Further, a persuasion index was created based on the primary outcomes. Only controlled or randomized-controlled studies were considered, and after screening, *K* = 39 of *N* = 4183 studies were included. Pairwise meta-analyses were conducted to determine the effects of EE on persuasive health outcomes. Additionally, potential effects of the subgroups *delivery mode*, *gender*, *healthcare, type of control group* and *type* and *taxonomy of health behavior* on a weighted persuasion index were analyzed. Analyses revealed evidence for significant small to moderate effects on all outcomes, with the largest effect sizes on knowledge and self-efficacy. Subgroups seem to account for a significant amount of heterogeneity, as a significant effect could be observed for all analyzed subgroups except for *healthcare* and *gender*. The meta-analysis suggests that EE is suitable for the communication of health messages, particularly when targeting prevention topics. However, it is imperative to exercise caution when interpreting the results, and further research is needed.

**Supplementary Information:**

The online version contains supplementary material available at 10.1038/s41598-025-11012-y.

## Introduction

Health communication is broadly defined as “any human communication about health”^1^. More specifically, strategic health communication—exemplified by health campaigns—adopts a targeted approach that aligns with persuasive communication, defined as the deliberate effort to influence attitudes, beliefs, or behaviors^2^. Within this framework, Schnabel^3^ characterizes health communication as “the use of as many target-oriented strategies as possible to reduce health risks and strengthen health resources.” By addressing psychological determinants such as attitudes, intentions, and perceived efficacy, health communication can effectively facilitate behavior change^4,5^. It is therefore evident that effective health communication plays a crucial role in the prevention of lifestyle-related issues and diseases, where behavioral adaptation represents an integral aspect^6,7^. Examples of such conditions include diabetes mellitus and cardiovascular diseases, which are influenced by behaviors such as physical inactivity, poor nutrition, and smoking^8–10^. However, existing models of health behavior suggest that increasing knowledge about health problems does not suffice to achieve a change in behavior. Instead, it appears to be necessary to affect parameters such as self-efficacy, attitudes, and intentions to achieve a sustainable adjustment of health behavior^11–14^. Thus, alternative, or complementary communication strategies instead of mere information transfer are likely to be required to effect a change in individual health behavior.

Entertainment-Education (EE) is a health communication strategy that has gained popularity rapidly^15^ and refers to “prosocial messages that are embedded into popular entertainment media content”^16^. Singhal and Rogers^17^ define EE as “the process of purposely designing and implementing a media message to both entertain and educate, in order to increase audience members’ knowledge about an educational issue, create favorable attitudes, shift social norms, and change overt behavior.“ However, existing literature exhibits conceptual ambiguity with regard to the implementation characteristics of EE. This pertains to the intended objective of the intervention, the utilization of comprehensive EE programs or discrete narrative strands, the context of implementation (natural or laboratory-based), and the attributes of the medium employed. The heterogeneity is reflected in the range of different examples of EE interventions: One common approach is to include health information in fotonovelas or soap operas, e.g. to reduce depression stigma^18^, or the infection rate of HIV^19^. In other studies, storylines were incorporated in popular TV series^20,21^, community theater^22^, or newspaper formats^23^ to exert a beneficial effect on health outcomes. To conclude, although EE encompasses a heterogeneous concept as regards health topics, target groups, channels, and practical implementation characteristics^15^, for the purpose of this study, we classify interventions as EE if they include both entertaining and educating elements and are embedded into popular media.

Various theories have been used to explain possible effects of EE. In the beginning, the *Social Cognitive Theory* was primarily applied as theoretical basis for the development of EE interventions, which assumes that the imitation of a character can cause adaptive health behavior^24,25^. Building on this, the *Extended Elaboration Likelihood Model*^26^ and *the Entertainment Overcoming Resistance Model*^16^ emphasize the potential of EE to reduce resistance against health promoting messages by evoking a more subliminal influence on recipients’ attitudes and intentions when compared with traditional health messages. In doing so, the models highlight the importance of message characteristics such as the quality of the production as well as media reception processes including absorption and identification^16^, which was mostly confirmed by empirical evidence^27,28^. Furthermore, both theoretical approaches and empirical evidence suggest that EE might be particularly useful for so-called “under-served populations”^29^. The term refers to “under-resourced” populations who have “limited access to services such as quality healthcare, education, healthy food sources, safe spaces, and other social determinants of health^30^”. EE might be suitable for these groups for several reasons: As this population group generally has a lower level of health literacy than the average population, narrative interventions could increase the comprehensibility of information^18,31^. Furthermore, the group’s accessibility could be enhanced by disseminating the messages via mainstream media, which are inherently capable of reaching a vast audience. The adaptation of narrative elements to align with the cultural norms and values of the target group has the potential to enhance the efficacy of the messages conveyed^18,32^. However, the range of use has widened over time and EE also seems to be effective for more educated individuals^33^. In general, EE is used in many different contexts today and it remains unclear which characteristics of the intervention and the target group increase the effectiveness of the intervention.

In order to synthesize the effects of EE and identifying potential moderator variables, several reviews and meta-analyses have been conducted. Primary reviews focused on theoretical conceptualizations of Entertainment-Education, such as Slater and Rouner^26^, and Moyer-Gusé^16,34^. A recent review investigated the effects of EE by including observational and experimental studies and found a small, but significant effect on persuasion^34^. Further, results pointed to a significant effect on knowledge, intentions, attitudes, and behavior. In addition, a significant effect of the subgroups *research design*, *exposure time*, and *gender* was detected, indicating a superior effect of EE in field studies, presented on multiple timepoints, and to men, respectively. By contrast, no significant effects were revealed for the following subgroups: *delivery channel*, *health issue* and *study location*.

In addition, various reviews focused on the comparative evaluation of narrative versus statistical evidence^35–37^. Narrative evidence differs from EE in that the message is not distributed through popular media, but other formats such as simple texts. However, existing research shows that an exact distinction remains difficult, as study authors define concepts differently.

In recent years, numerous studies investigating the effects of EE have been published^38–40^, requiring an updated synthesis of study results with a clear definitional scope. As the review of Shen and Han^34^ included mostly observational studies, a meta-analysis only considering controlled and randomized-controlled trials could give more insight into causal effects of EE interventions. In addition, replicating the investigation and including further potential moderator variables could enhance the understanding of the underlying mechanisms of EE and advance theoretical approaches.

Thus, the current systematic review and meta-analysis aims to give an updated overview of the effects of EE on persuasive health outcomes. For this purpose, a broad definition of EE is used, also including interventions implemented in media developed by the study authors and campaign practitioners (such as videos), and in laboratory settings. Further, moderator variables for the potential effects of EE are investigated. For the derivation of these variables, we adapt the subgroups of Shen and Han^34^, and additionally apply the Lasswell scheme^41^; see Supplementary Table 1], which categorizes the process of communication into the following characteristics: source, message, channel, and recipient. Based on the findings, implications for the design of Entertainment-Education interventions and future research will be deduced.

## Material and methods

The current systematic review and meta-analysis was registered in PROSPERO (registration number: CRD42020211662) and is reported in accordance with the Preferred Reporting Items for Systematic reviews and Meta-analysis (PRISMA statements)^42^.

### Search strategy

The following databases were searched until January 31, 2024 to identify relevant studies for the meta-analysis: MEDLINE, PsycINFO/PsycARTICLES, Web of Science and ClinicalTrials.gov (see Appendix A for Pubmed search strategy). We included English peer-reviewed publications and applied no time restrictions. The search strategy combined the following search blocks by using the Boolean operators “AND” and “OR”: (1) Health communication and related constructs (e.g., health promotion, health education), (2) intervention (e.g., entertainment-education, drama, narration) (3) outcomes (e.g., health knowledge, health attitudes), (4) study design (e.g., randomized-controlled trial, controlled clinical trial). When possible, study design restrictions were also adjusted by using the filter functions of the databases. In addition, the references of the included studies were checked for further relevant trials and primary studies of the reviews of Shen and Han^34^, and Shen et al.^36^ were screened for eligibility. Beyond that, the primary investigator checked further studies known through experience in this research field.

### Eligibility criteria

Studies were included if they fulfilled the following criteria: (1) Study participants are ≥ 18 years old; (2) the study includes an intervention which deals with a health-relevant topic (prevention, detection, diagnosis,treatment, survivorship,end-of-life); (3) the intervention consists of entertaining (e.g., use of drama, humor) and educating (e.g., knowledge sharing, problematization) components as well as using a popular medium (e.g., radio, theater). As we aimed to include controlled studies and randomized-controlled studies, we chose a broad conceptualization of EE, also considering interventions implemented in own (popular) media and investigated in laboratory settings. By contrast, studies which used the terms “message”, “story”, or “narratives” were excluded^43,44^ to ensure the conceptual distinction between EE and narrative evidence; (4) the EE intervention is compared to a purely informative intervention, attention control group, waitlist control group, or no intervention; (5) the study has a controlled, randomized-controlled, cluster-randomized, or crossover design; (6) based on the definitions of Shen and Han^34^, the study includes one or more of the following outcomes: knowledge, attitude, intention, behavior. In light of the extant conceptual framework, “persuasive health outcomes” are defined here as follows: Mental states that function as precursors of an intended alteration in health behavior^5,45^.

### Screening

**Fig. 1:**
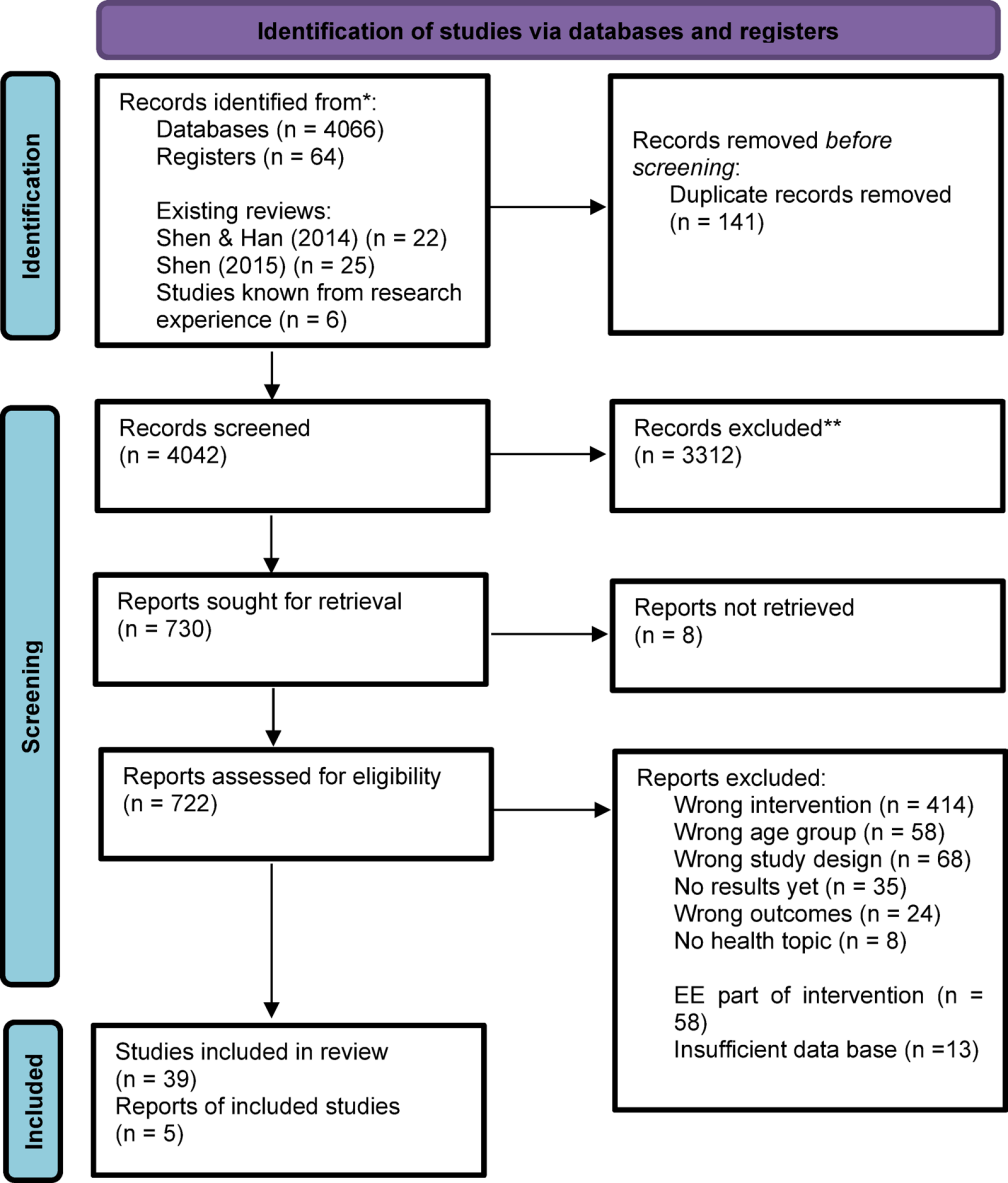
Flow diagram. Created based on Page et al.^42^.

The screening process was done using the tool Covidence, developed by the Cochrane Collaboration^46^. For this purpose, identified references were imported into Covidence which removed duplicates automatically. Title and abstract of imported references were checked by two independent raters, and irrelevant studies were excluded. In the next step, all remaining studies were reviewed for eligibility at full-text level and inappropriate studies were excluded by stating an exclusion reason. At both screening levels, disagreements between reviewers were resolved by discussion. Abstracts of references of Shen and Han^34^, Shen et al.^36^, and studies known by research experience were checked by one reviewer and included into the review process if the criteria seem to be fulfilled. The flow of studies is depicted in Fig. 1.

### Data extraction and coding

Extracted data included the following information: Study characteristics (e.g., country, funding), methods (e.g., research design, level of randomization), population (e.g., sample size, eligibility criteria, demographic characteristics), intervention (short description of the intervention, theoretical basis) and primary and secondary outcomes as well as their measures. Based on the existing meta-analysis on the effects of EE^34^, primary outcomes included health knowledge, health attitudes, health intentions, and health behavior. Most secondary outcomes were derived from existing models of health behavior^11–14^, and comprised self-efficacy, outcome expectations, perceived risk, subjective norms, perceived vulnerability, perceived costs, perceived benefits, perceived severity, perceived barriers, and perceived resources. To achieve a broader understanding of possible effect mechanisms, secondary outcomes included satisfaction with, evaluation and acceptance of and adherence to the intervention, if applicable. Subgroups were developed based on the findings of Shen and Han^34^ and results of the above-described studies. Furthermore, subgroups were derived from theoretical approaches^16,26^ and from a literature review, taking into account frequently assessed characteristics of studies investigating EE. All subgroups were categorized within the Lasswell scheme^41^. In doing so, the subgroup *taxonomy of health behavior* is predicated on the assumption that there is a consistent internal organization to the clustering of health behaviors^47^, which extends beyond the classification of prevention, detection, or cessation behavior, as organized here in *type of health behavior*. More specific, we based the categories of the subgroup *taxonomy of health behavior* on a study, which clustered 45 health behaviors into the following categories: general well-being (e.g., holidays), risk avoidance (e.g., smoking), nutrition (e.g., fresh food) and health maintenance (e.g., vaccinations) which saved as basis for the subgroup categorization^47^.

### Quality assessment

The Cochrane Collaboration’s tool for assessing risk of bias was used to evaluate the quality of the included studies^48^. The tool includes the following domains: *sequence generation*, *allocation concealment*, *blinding of participants and personnel*, *blinding of outcome assessment*, *incomplete outcome data*, and *selective outcome reporting*. The *categories blinding of participants and personnel* and *blinding of outcomes assessment* were assessed separately for subjective and objective outcomes (behavioral outcomes that were not measured via self-reporting). As we also included cluster-randomized and controlled trials, we added the domain *baseline comparability* of experimental groups. Two independent raters coded each domain as low risk (0), unclear risk (1), or high risk (2). A sum score across the nine domains was calculated to assess an overall risk of bias for each study.

### Data analysis

The data analysis was carried out in a multi-staged process. Initially, a summary effect statistic (*SMD*, 95% *CI*) for each study on prespecified primary and secondary outcomes was calculated, if applicable. Effect sizes that were stated within a primary study were extracted, if appropriate. When both active and passive control groups were reported, we chose a conservative approach by including the data of the active control group.

In case of missing data, study authors were contacted and otherwise data was imputed building on the recommendations of the Cochrane Collaboration^49,50^. To aggregate data of multiple subgroups, we averaged sample sizes, means and standard deviations based on the references of the Cochrane Collaboration^49^. If an outcome was measured by multiple instruments, we calculated a weighted effect size, as recommended by Borenstein et al.^51^. As the correlations between dependent outcomes are mostly unknown, we chose a conservative approach by using correlations of *r* = 1. After determining the effect size for each primary and secondary outcome on study level, we calculated a weighted persuasion index. The utilization of an index is a prevalent practice when synthesizing the persuasive effects of health communication interventions^52,53^. Specifically, the persuasion index of the current meta-analysis was based on the existing meta-analysis on the effects of EE^34^, and reflects the averaged effect of the primary outcomes. In addition, theoretical conceptualizations were considered, such as the definition by Ort and Fahr^2^, which posits that persuasive communication “aims to stimulate, develop, stabilize, or change individuals’ health-related knowledge, attitude, or behavior.” Miller^54^ likewise emphasizes that persuasion can target various types of responses, including intentions and behavior. In order to aggregate the primary outcomes to the weighted persuasion index, we converted dichotomously measured data into continuous data, building upon the guidance of the Cochrane Collaboration^50^. On this basis, we determined a combined intervention effect for each primary and secondary outcome as well as the persuasion index by applying pairwise meta-analysis. The Grubbs test^55,56^ was used to identify outliers across the effect sizes. The Grubbs test checks the value in a dataset that is furthest from the mean. It calculates a test statistic by taking the absolute difference between that value and the mean, divided by the standard deviation. This statistic is then compared to a critical value at a chosen significance level (e.g., α = 0.05) to determine whether the value is a statistical outlier.

To interpret the effect sizes, the guidelines of Cohen^57^ were used. We calculated confidence intervals and *p* values and studied heterogeneity between primary studies by assessing *I*^2^. *I*^2^ was interpreted in accordance with the guidelines of the Cochrane Collaboration^50^. Given that EE is a heterogeneous concept, we applied the random-effects model. In addition, we conducted subgroup analyses to find possible explanations for heterogeneity between groups. Interrater reliability was determined by the coefficient Cohen’s Kappa^58,59^. To assess publication bias, the funnel plot was visually inspected, and Egger’s test^60^ was conducted. The Python command used for Egger’s test^60^ was created with the assistance of OpenAI’s ChatGPT^61^ and executed in a Google Colaboratory environment^62^ using Python 3.10^ 63^. Other analyses were done using the Software RevMan^64^.

### Post protocol changes

Some post protocol data analysis modifications were made due to the revealed data situation. The screening process disclosed that some of the studies examined interventions including EE as well as other components. In order to draw strong conclusions, we decided to exclude studies involving interventions with EE and additional components (see Fig. 1). In terms of analyzed timepoints of the primary outcomes, we calculated the averaged effect for both timepoints (post, follow-up) instead of an effect size for every timepoint. By contrast, we determined the effect size for the weighted persuasion index separated for each timepoint (post, follow up) as well as an effect size averaged over both timepoints. Contrary to the other included studies, Hernandez and Organista^65^ reported change-of-baseline data instead of post-intervention values. As a review cited by the Cochrane Collaboration^50,66^ found no differences in results when combining both data types, we decided to include this study. During the screening process, we discovered that some studies measured stigma as an outcome and decided to include it into the outcome attitude by inverting the sign. To clarify, the inclusion was based on a definition of stigma highlighting its conceptual similarity to attitudes: stigmatization can be defined as negative attitudes and beliefs, which may be either conscious or unconscious^67,68^. For instance, numerous studies have investigated stigmatizing attitudes within the contexts of mental health^69,70^ and sexual health^71^.

Moreover, self-efficacy was the only pre-specified secondary outcome revealing a sufficient data base and thus the remaining secondary outcome data was not analyzed. Furthermore, we only included the following subgroups into analyses, due to little variance in the remaining pre-specified subgroups: *delivery mode*, *gender*, *type of health behavior*, *taxonomy of health behavior*. In order to establish a subgroup that reflects the under-served population, the decision was made to combine the pre-specified subgroups *setting* and *education* into a subgroup called *healthcare*. This decision was based on literature^72^. More specific, studies incorporating participants with a migrant background, which is indicative of disadvantages with regard to healthcare, participants with low income, and participants residing in Low -and Middle-income Countries were coded as “under-served”. Conversely, studies that did not fulfil these criteria were classified as “not under-served “. Beyond that, we chose to include *type of control group* (active vs. passive) as an additional post hoc subgroup to get more insights into relevant moderator variables (see Supplementary Table 2).

## Results

### Study flow

After searching the above-mentioned databases, *N* = 4,130 studies were included into the screening process in Covidence (Fig. 1). Additional *N* = 53 studies were incorporated from the above-mentioned external sources. Of the total *N* = 4,183 studies, *N* = 141 duplicates were removed. After screening title and abstract, *N* = 3,312 studies were excluded. The remaining studies were screened at a full-text level, and further *N* = 678 studies were sorted out. The main exclusion reason was that the respective study did not investigate EE as an intervention. As *N* = 5 studies were reports of the same data, the final sample size comprised *N* = 39 studies.

### General study data

Most of the primary studies were conducted in the U.S. (*k* = 27). The remaining studies took place in the following countries and regions: Austria, China, Germany, Nepal, Nigeria, South Africa, the Netherlands, and Taiwan. Beach et al.^73^ did not provide the number of randomized participants. Similarly, no information could be obtained for participants ≥ 18 years which were included from Davis and Jansen^74^, and Saucier et al.^75^. Following, this resulted in a summarized *N* = 12,387 across the remaining studies.

The mean indicated age ranged from *M* = 19.72 (*SD* = 1.32) to *M* = 60.5 (*SD* = 12.0) years. 70.4% of participants across calculable studies identified as female. In terms of education, *k* = 10 studies included participants with at least a high school degree, while *k* = 23 studies considered individuals with different education levels and *k* = 6 studies did not provide any information. An overview of the general study data is provided in Supplementary Table 3.

### Effects of EE on health outcomes

#### Knowledge

**Fig. 2:**
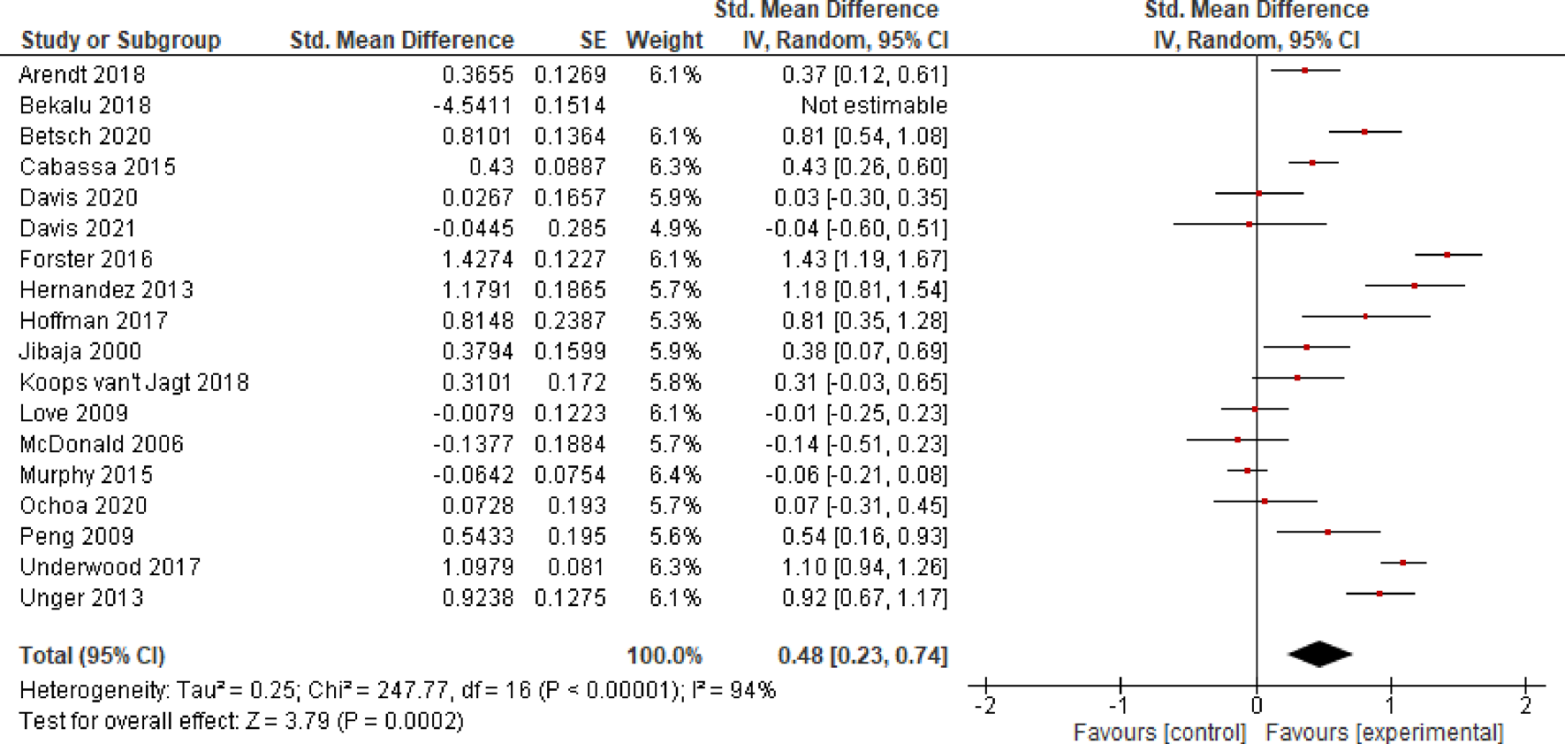
Effects of Entertainment-Education on knowledge.

After aggregating the effects of *k* = 18 studies, Bekalu et al.^76^ was identified as an outlier, and analysis including this study yielded no evidence for a significant effect (*SMD* = 0.20, *CI* = − 0.34–0.74, *p* = 0.470). The exclusion of Bekalu et al.^76^ resulted in evidence for a significant moderate effect of EE (*SMD* = 0.48, *CI* = 0.23–0.74, *p* < 0.001). However, as the confidence interval is large, the determination of the true effect is imprecise. The coefficient *I*^2^ (94%) indicates a considerable heterogeneity between studies (Fig. 2).

#### Intention

**Fig. 3:**
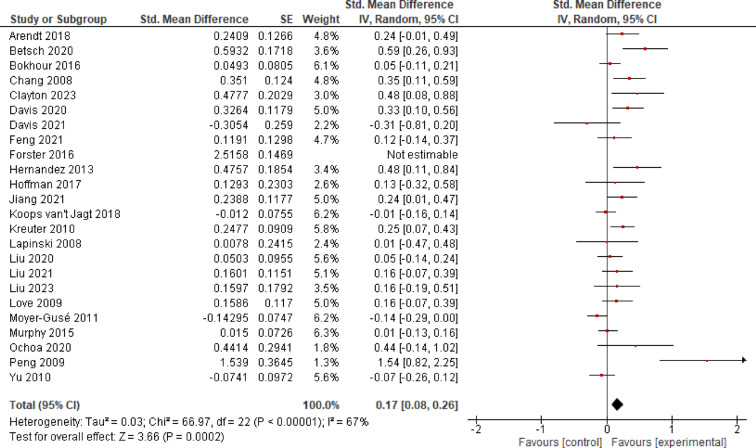
Effects of Entertainment-Education on intention.

*K* = 24 studies investigated the effects of EE on health intentions. After removal of the outlier Forster et al.^77^, the overall effect size diminished from *SMD* = 0.30 (*CI* = 0.12–0.49) to *SMD* = 0.17 (*CI* = 0.08–0.26), indicating evidence for a significant small effect (*p* < 0.001). *I*^2^ was 67%, which corresponds to substantial heterogeneity between studies (Fig. 3).

#### Attitude

**Fig. 4:**
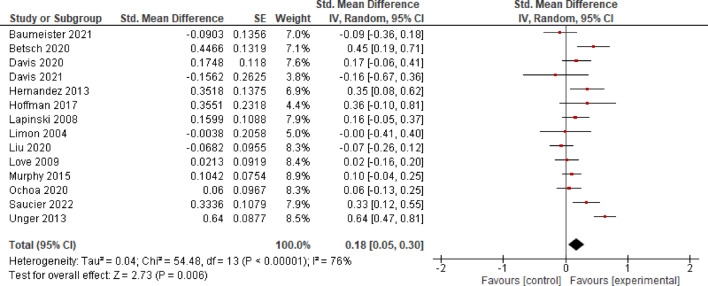
Effects of Entertainment-Education on attitude.

*K* = 14 studies were included to determine the effect of EE on health attitudes. *K* = 3 of these studies measured the stigma towards a particular health topic. Analysis revealed evidence for a significant small effect (*SMD* = 0.18, *CI* = 0.05–0.30, *p* = 0.006), pointing to a considerable amount of heterogeneity (*I*^2^ = 76%; Fig. 4).

#### Behavior

**Fig. 5:**
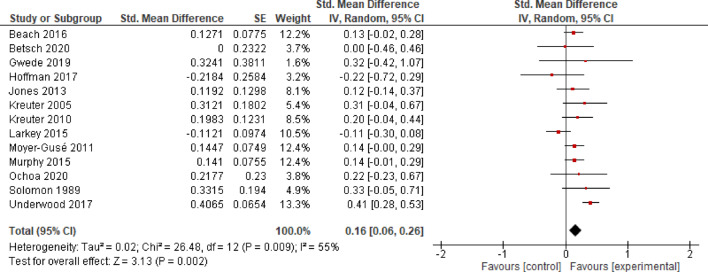
Effects of Entertainment-Education on behavior.

*K* = 13 studies were considered to analyze the effects of EE on health behavior. The analysis revealed an effect size of *SMD* = 0.16 (*CI* = 0.06–0.26), indicating evidence for a significant small effect (*p* = 0.002) and moderate heterogeneity (*I*^2^ = 55%; Fig. 5).

#### Self-efficacy

**Fig. 6:**
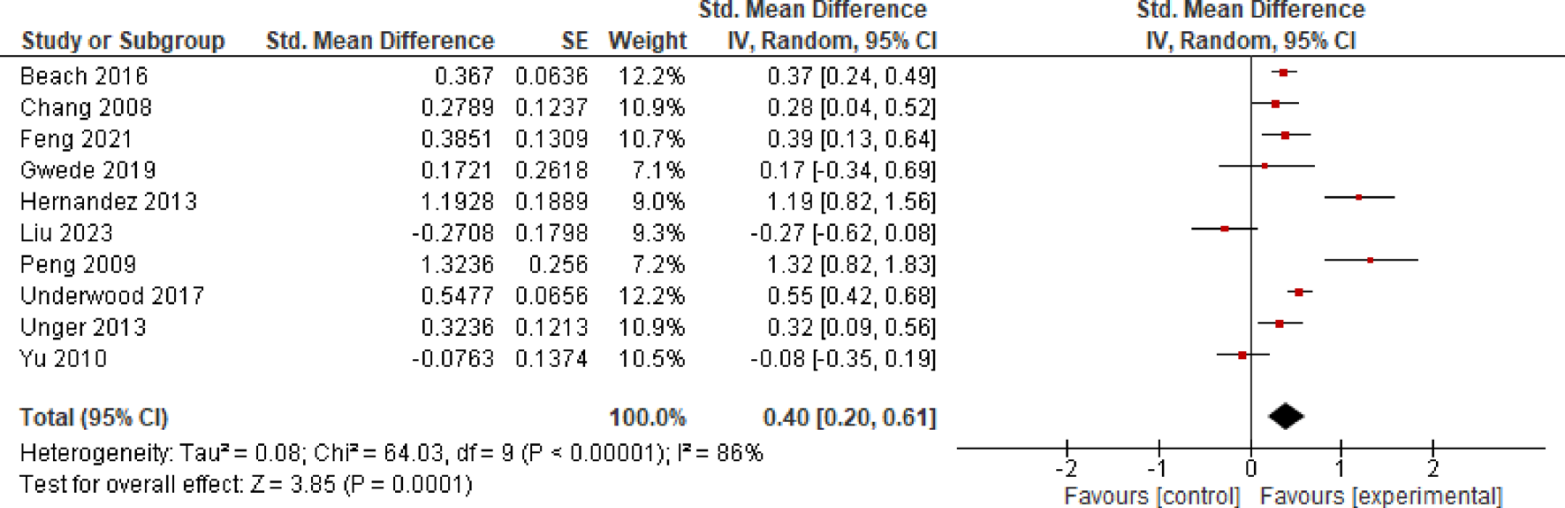
Effects of Entertainment-Education on self-efficacy.

*K* = 10 studies investigated the effects of EE on self-efficacy. The analysis yielded evidence for a significant moderate effect of *SMD* = 0.40 (*CI* = 0.20–0.61, *p* < 0.001), and a considerable heterogeneity between studies (*I*^2^ = 86%). The width of the confidence interval suggests that the true effect is not precisely known (Fig. 6).

#### Weighted persuasion index

The initial analysis including the outlier Bekalu et al.^76^ yielded no evidence for a significant effect on persuasion at the post timepoint (*k* = 30, *SMD* = 0.16, *CI* = − 0.11–0.42, *p* = 0.250). Conversely, after excluding this study, the analysis revealed evidence for a significant small effect of *SMD* = 0.32 (*k* = 29, *CI* = 0.15–0.48, *p* < 0.001) on persuasion at the post timepoint, and a considerable heterogeneity between studies (*I*^2^ = 95%; Supplementary Fig. S1).

The averaged effect size for the follow-up timepoint (*k* = 17) suggests evidence for a significant effect (*SMD* = 0.23, *CI* = 0.01–0.46, *p* = 0.040), and considerable heterogeneity between studies could be observed (*I*^2^ = 98%; Supplementary Fig. S2).

**Fig. 7:**
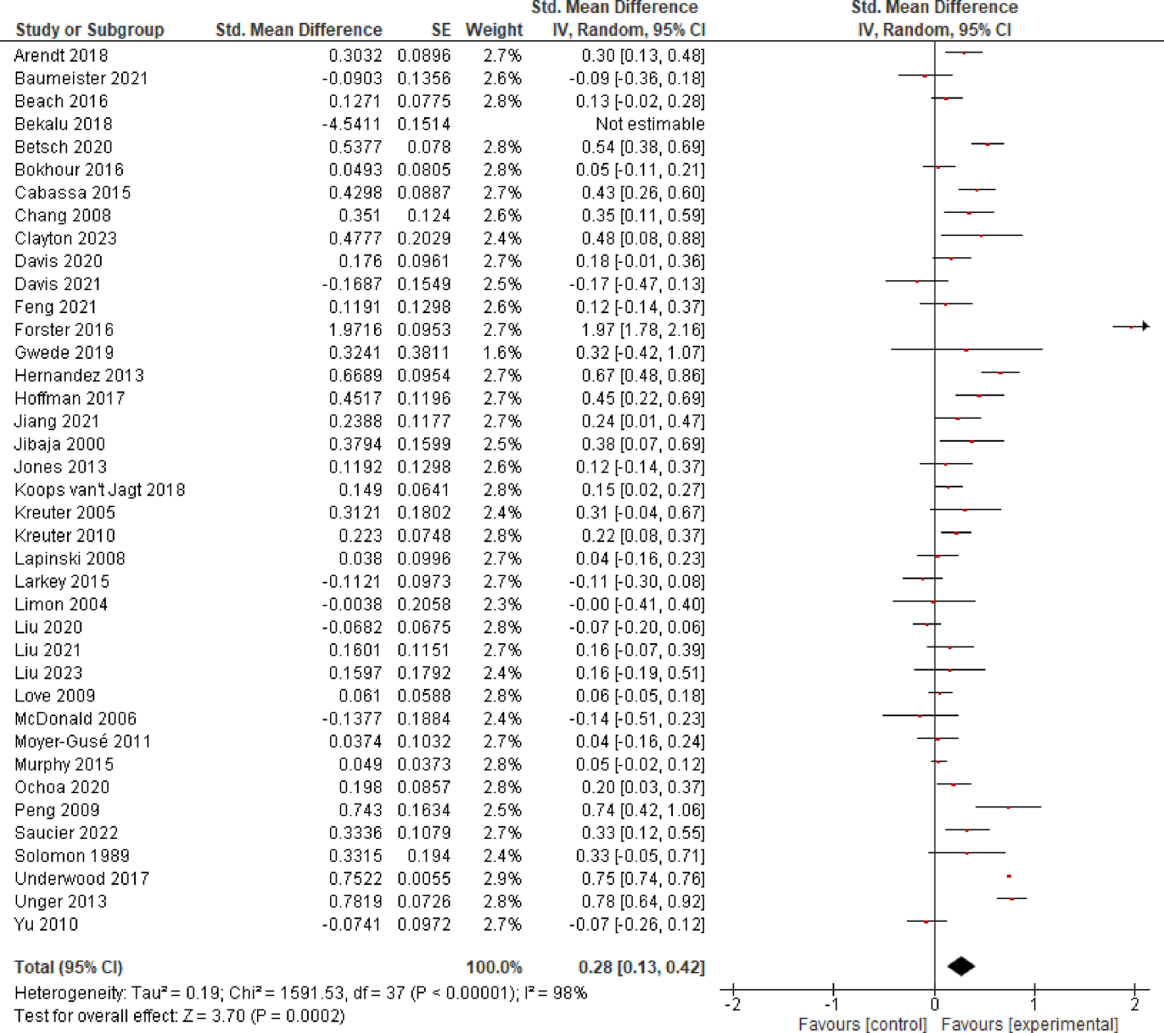
Effects of Entertainment-Education on persuasion.

Including the outlier Bekalu et al.^76^, no evidence for a significant effect on persuasion for the combined timepoints could be observed (*SMD* = 0.15, *CI* = − 0.03–0.34, *p* = 0.110). By contrast, after removing Bekalu et al.^76^, analysis revealed evidence for a significant small effect of *SMD* = 0.28 (*CI* = 0.13–0.42, *p* < 0.001). Results point to a considerable heterogeneity between studies (*I*^2^ = 98%; Fig. 7).

### Subgroup analyses

Subgroup analyses were conducted for the combined weighted persuasion index. Analyses revealed a significant effect for the subgroups *type of control group* (χ(1)^2^ = 4.84, *p* = 0.030; Supplementary Fig. S3), *taxonomy of health behavior* (χ^2^(4) = 17.31, *p* = 0.002; Supplementary Fig. S4), *type of health behavior* (χ^2^(2) = 7.41, *p* = 0.020; Supplementary Fig. S5), and *delivery mode* (χ^2^(2) = 20.07, *p* < 0.001; Supplementary Fig. S6). The studies including *passive control groups* (*SMD* = 0.60) showed a superior effect on persuasion compared to *active control groups* (*SMD* = 0.19). Investigating the subgroup *taxonomy of health behavior* descriptively, studies examining the topics *nutrition* (*SMD* = 0.53) and *general well-being* (*SMD* = 0.51) revealed the largest effect sizes. Studies including *health maintenance (SMD* = 0.29) and *mixed topics* (*SMD* = 0.32) showed small effect sizes, whereas studies examining *risk avoidance* (*SMD* = 0.09) did not obtain any effect. Regarding the subgroup *type of health behavior*, the effect size associated with *prevention* (*SMD* = 0.32) is descriptively larger than that associated with *detection* (*SMD* = 0.15) and *cessation* (*SMD* = − 0.01). In the subgroup *delivery mode*, the largest effect is demonstrated descriptively for the *mixed group* (*SMD* = 0.63), followed by *video* (*SMD* = 0.24). The subgroups *gender* (χ^2^(1) = 0.01, *p* = 0.930; Supplementary Fig. S7), and *healthcare* (χ^2^(1) = 3.14, *p* = 0.080; Supplementary Fig. S8) did not yield any significance. However, a tendency was observed that the studies examining under-served groups exhibited a higher descriptive effect size compared to those involving non-under-served participants.

### Quality assessment

The quality of the primary studies ranged between 1 and 11 points, and the mean for the overall study quality was *M* = 5.36 (*SD* = 6.24; Supplementary Figs. S9 and S10). Only *k* = 4 studies included objective measures, thus reducing the significance of the results regarding the domains *blinding of participants and personnel* and *blinding of outcome assessment*. By contrast, the domains *blinding of participants and personnel* and *blinding of outcome assessment* for subjective outcomes showed a particular high risk for bias, whereas the domains *incomplete outcome* and *baseline comparability* revealed an unclear risk for many studies.

### Interrater reliability

The averaged interrater reliability between the three reviewers was κ = 0.53 for the screening of title and abstract and κ = 0.57 for the screening at full text level, indicating a moderate agreement^59^.

### Publication bias

The funnel plot exhibits an asymmetrical distribution of studies around the vertical line representing the overall effect size, with a greater number of studies positioned to the left of this line compared to the right (Supplementary Fig. S11). Further, the majority of studies are concentrated towards the top of the plot, with standard errors between 0 and 0.2, indicating that most studies are of high precision. The statistical examination of funnel plot asymmetry using Egger’s regression test^60^ provides further evidence of potential publication bias in the meta-analysis, as indicated by a significant result (*t*(36) = − 5.57, *p* < 0.001).

## Discussion

The present systematic review and meta-analysis aimed at giving an updated overview of the effects of EE on persuasive health outcomes by considering various moderator variables. Our results indicate evidence for significant small to moderate effects of EE on the weighted persuasion index as well as on primary outcomes and self-efficacy^57^. However, it must be considered that the confidence intervals of some outcomes are wide, which makes it difficult to determine the effects’ precision. Besides, the confidence intervals of several outcomes almost overlap nil, questioning the practical relevance of the underlying effect. In general, *I*^2^ values point to a huge heterogeneity between primary studies. *Taxonomy of health behavior, type of health behavior, delivery mode,* and *type of control group* are significant subgroups for the effect on persuasion, whereas no effect could be observed for *gender* and *healthcare*.

Indicating evidence for a significant effect of EE on persuasion and primary outcomes, the current findings are congruent with results of the existing review on effects of EE^34^. In general, the present study found descriptively higher effect sizes than Shen and Han^34^. This could be explained by the fact that the present study only included studies which were at least controlled, thereby eliminating potentially confounding variables occurring in observational research designs. However, this finding is incongruent with the subgroup analysis of Shen and Han^34^, which indicated that field studies investigating EE showed superior effects compared to experimental studies. An alternative explanation may be that, in naturalistic settings, the implicit persuasive mechanisms proposed in EE theories^16,26^—such as narrative transportation and reduced counterarguing—are more likely to be activated and exert their intended effects. These mechanisms depend on the audience’s emotional and cognitive engagement with the narrative, which may be more effectively facilitated in real-world settings. By contrast, laboratory settings may constrain such immersion due to the artificiality of the environment or participants’ awareness of being observed, thereby attenuating the persuasive impact of the entertainment content. Given the theoretical plausibility of both interpretations, further empirical research is needed to systematically examine the influence of contextual factors on the effectiveness of EE.

Further, the findings replicate the highest effect for knowledge in comparison with the other primary outcomes, although effects sizes were only contrasted descriptively. Shen and Han^34^ concluded that knowledge pertains to proximal responses to message exposure. Proximal responses can be affected more easily as they are cognitive-based and thereby activated prior to more distal reactions such as intention, attitude and behavior^34,78^. Theoretically, it may take less effort for recipients to pay attention to a message with additional entertaining components and thus to absorb arguments better than in purely informative interventions.

The relatively modest effect size observed for behavioral outcomes may be attributed to theoretical frameworks that posit behavioral change occurs only after psychological variables have been adapted^11,14^. In EE, this could be knowledge and self-efficacy, whereby attitudes and intention did not achieve higher effect sizes than behavior, as would be expected. However, regarding the outcome health attitude, it must be considered that health stigma has been included, thus possibly reducing the validity of its measurement. Although both concepts include an evaluation component, health stigma possibly entails a higher personal threat and is associated with feelings of shame^79,80^. As part of a meta-analysis, Zebregs et al.^37^ found narrative evidence to be more effective in changing health intentions, and reversely statistical evidence in influencing health attitudes. As the current meta-analysis did not replicate this finding, it questions the authors’ conclusion that intentions have a greater emotional component and are influenced to a greater extent by narrative interventions, whereas attitudes include a more rational component and are thus affected more by informational interventions. As previously stated, the utilization of narrative evidence and EE varies with respect to the conceptual components involved.

In addition to the review of Shen and Han^34^, self-efficacy was included as an outcome and reveals evidence for a significant medium effect size. This finding could be explained by the *Social Cognitive theory* which states that individuals acquire new behavior by observing models^24,25^. In doing so, the theory highlights the importance of self-efficacy for behavioral adjustment^81^.

In contrast with Shen and Han^34^, the current meta-analysis found a significant moderator effect for the subgroups *delivery mode* and *type of health behavior.* Specifically, both subgroup analyses *type of health behavior* and *taxonomy of health behavior* indicate a superior effect of EE for preventive health behaviors as compared to risk-associated behaviors such as detection examinations or substance abuse^47^. Differences in the effectiveness of communication strategies for prevention and detection behavior have also been established in other contexts^53,82^, indicating a conceptual difference between both types of behavior. Considering the present analysis, it is possible that the entertaining elements of EE are perceived as not suitable for potentially threatening health topics. Preventive health topics often resonate with a broad audience, as they address widely relevant issues and are typically not perceived as threatening. This can increase openness and message elaboration, as explained by the *Elaboration Likelihood Model*^83^, which suggests that peripheral cues can enhance message processing. Moreover, the anticipated enjoyment of entertaining content may align with the positive emotions often associated with preventive health topics.

In contrast, EE interventions on risk-associated health topics may elicit defensive responses due to their connection with negative emotions such as fear or shame^84,85^. According to *Social Cognitive Theory*^24,25^, media characters in EE narratives could serve as role models and promote learning through imitation—a mechanism potentially more effective in the context of preventive health than with high-risk topics. While humor is frequently employed in EE formats, it can prove counterproductive in high-risk contexts by attenuating the perceived seriousness of the message and diminishing its persuasive impact, particularly among audiences personally affected^86,87^.

The superior effect of subgroup *other, multimodal, audio* in comparison to the others may be attributed to the observation that vivid representations addressing different perceptual modalities^22,33^ tend to be more convincing^88^. However, it is imperative to exercise caution in the interpretation of these findings, given that a mixed category was formed due to the limited number of studies included in the analysis. Additionally, although no significant effect could be observed for the subgroup *healthcare*, descriptive results indicate that EE may be particularly effective for under-served populations. This finding is consistent with theoretical assumptions which postulate that EE can overcome barriers to understanding of purely informative health messages, e.g. low levels of health literacy^18,31^. One reason why the health subgroup was not found to be significant may be that the groups based on the PROGRESS factors^72^ were not clearly delineated. It should also be noted that some studies included participants with variable characteristics (e.g. cultural background), which further complicates the distinction.

The meta-analysis dealt with common limitations of reviews in general, including language bias due to the inclusion of only English literature, publication bias due to the exclusion of “grey literature” and the screening of a limited amount of data bases^89,90^. Identifying relevant studies posed a particular challenge due to the multidisciplinary nature of EE^91^. Consequently, individual databases specific to each discipline involved (e.g., communication science, media science) were not searched separately. The inclusion of references of additional sources potentially increased the risk of bias. A methodological limitation of particular concern is the so-called garbage-in-garbage-out problem, referring to the increase of methodological shortcomings due to combining them^51^. The results of the quality assessment indicated a limited quality of some studies, which could serve to exacerbate the garbage-in-garbage-out problem. In addition, missing data was a frequent problem for the present meta-analysis, which resulted in the inability to perform all pre-specified analyses. Although imputation techniques were based on the guidelines of the Cochrane Collaboration^49,50^, some data problems evolved which had to be solved with divergent solutions, possibly inducing a bias. Furthermore, the “combination of apples and oranges” is a controversial topic in the context of meta-analyses and describes the incidence of methodological problems due to synthesizing heterogenous studies in one analysis^92^. This aspect could be especially relevant for the current analysis due to a lack of clarity in the literature regarding the definition of EE. Although we applied a clear definitional scope within the current analysis, definitional ambiguity in existing literature could be one reason for the modest interrater reliability in the screening process and is potentially reflected in the partially high *I*^2^ values.

The current meta-analysis reveals gaps in the research field of EE. In general, more studies with a controlled and randomized-controlled are needed. For this purpose, measures for relevant persuasive outcomes regarding different health topics should be developed and validated. Furthermore, self-efficacy should be included as an outcome in prospective investigations as both theoretical approaches and current results emphasize its relevance for the effects of EE. Moreover, future research should investigate additional variables that may influence the effectiveness of EE. For the purpose of classification, Lasswell’s communication model^41^ may serve as a useful framework, distinguishing between the categories of source, message, channel, and recipient. For example, further demographic variables related to the recipient—such as age—could be examined more systematically. In general, it is crucial to address the conceptual ambiguity regarding the definition of EE. Particularly in terms of implementation features a consistent operationalization must be reached to enable meaningful research and to allow for reaching clear conclusions.

The current meta-analysis gives an updated synthesis of the effects of the health communication strategy EE, thus contributing to the understanding of underlying mechanisms. Findings point to the effectiveness of EE on persuasive health outcomes. EE seems to be a particularly suitable approach if intervention planners aim to increase recipients’ knowledge and self-efficacy and to address prevention topics. Given its considerable practical relevance, a central objective is the development of guidelines which include scientifically substantiated recommendations for practitioners. However, as current findings are based on a limited data base, more research in the domain is needed.

## Electronic supplementary material

Below is the link to the electronic supplementary material.

Supplementary Material 1

## Data Availability

The datasets generated and/or analyzed during the current study will be made available in the Zenodo repository upon publication of this article. The persistent web link and accession number will be provided at that time. Further queries/data available from the corresponding author on reasonable request.
