# Supplementary material for: A systematic review and meta-analysis of the effects of Entertainment-Education interventions on persuasive health outcomes
Source: Sci Rep. 2025 Jul 31;15:27939. doi: 10.1038/s41598-025-11012-y (PMC12314045; doi:10.1038/s41598-025-11012-y)
Supplement: Supplementary file 1 — Supplementary Material 1 [file 41598_2025_11012_MOESM1_ESM.docx]

Supplementary Table 1. Coding system of planned subgroup analyses.

| Sub Group | Coding |
| --- | --- |
| Source |  |
| Originator of the message | 1 = Health service  2 = Popular entertainment media |
| Message |  |
| Type of health behaviour | 1 = Prevention  2 = Detection  3 = Cessation  4 = Other |
| Taxonomy of health behaviour | 1 = Health maintenance  2 = Nutrition  3 = Risk avoidance  4 = General well-being  5 = Mixed  6 = Other |
| Setting | 1 = LMIC  2 = Industrialized Country |
| Aim of the intervention | 1 = Dedicated intervention  2 = General media effects |
| EE-strategy | 1 = Humor  2 = Drama  3 = Testimonial  4 = Other  5 = Mixed |
| Theoretical basis | Open text box |
| Exposure time | 1= Once  2 = More than once |
| EE Info part | 1 = Embedded  2 = Separated |
| Canal |  |
| Delivery mode | 1 = Audio  2 = Video  3 = In writing  4 = Multimodal  5 = Other |
| Channel | 1 = Radio  2 = TV  3 = Online/Website  4 = Article/Newspaper/Magazine  5 = Comic  6 = Theater  7 = Games  8 = Other |
|  |  |
| Recipient |  |
| Gender | 1 = Men  2 = Women  3 = Mixed gender |
| Age | Mean of the sample |
| Education | 1 = Mixed  2 = Open text box |
| Directedness | 1 = Direct Recipient  2 = Caregiver |
| Other |  |
| Research design | 1 = Randomized controlled trial  2 = Cluster randomized trial  3 = Controlled trial  4 = Crossover trial |

Supplementary Table 2. Subgroup categorization for included studies.

| Study | Delivery mode | Gender | Taxonomy of health behavior | Type of health behavior | Control group | Healthcare |
| --- | --- | --- | --- | --- | --- | --- |
| Arendt 2018 | Writing | Mixed | Mixed | Prevention | Passive | Not U. |
| Baumeister 2021 | Video | Mixed | HM | Prevention | Active | Not U. |
| Beach 2020 | Video | Mixed | HM | Prevention | Active | Not U. |
| Bekalu 2018 | Video | Mixed | HM | Prevention | Active | Not U. |
| Betsch 2020 | Audio | Mixed | HM | Prevention | Active | Not U. |
| Bokhour 2016 | Video | Mixed | Mixed | Prevention | Active | Under-served |
| Cabassa 2015 | Writing | Mixed | GWB | Prevention | Active | Under-served |
| Chang 2008 | Writing | Mixed | GWB | Prevention | Active | Not U. |
| Clayton 2023 | Video | Mixed | RA | Prevention | Active | Not U. |
| Davis 2020 | Writing | Mixed | RA | Prevention | Active | Under-served |
| Davis 2021 | Writing | Mixed | RA | Prevention | Active | Under-served |
| Feng 2021 | Video | Mixed | RA | Cessation | Active | Not U. |
| Forster 2016 | Video | Mixed | HM | Prevention | Passive | Under-served |
| Gwede 2019 | Other | Mixed | HM | Detection | Active | Under-served |
| Hernandez 2013 | Other | Female | GWB | Prevention | Active | Under-served |
| Hoffman 2017 | Video | Mixed | HM | Detection | Active | Under-served |
| Jiang 2021 | Video | Mixed | HM | Prevention | Active | Not U. |
| Jibaja 2000 | Other | Female | HM | Detection | Active | Under-served |
| Jones 2013 | Video | Female | RA | Prevention | Active | Under-served |
| Koops van’t Jagt 2018 | Writing | Mixed | Mixed | Prevention | Active | Not U. |
| Kreuter 2010 | Video | Female | HM | Detection | Active | Under-served |
| Kreuter 2005 | Writing | Female | Nutrition | Prevention | Passive | Under-served |
| Lapinski 2008 | Video | Mixed | RA | Prevention | Passive | Under-served |
| Larkey 2015 | Video | Mixed | HM | Detection | Active | Under-served |
| Limon 2004 | Video | Mixed | RA | Prevention | Active | Not U. |
| Liu 2020 | Writing | Mixed | RA | Cessation | Active | Not U. |
| Liu 2021 | Writing | Female | HM | Prevention | Active | Not U. |
| Liu 2023 | Video | Mixed | GWB | Prevention | Active | Not U. |
| Love 2009 | Video | Female | HM | Detection | Active | Under-served |
| McDonald 2006 | Writing | Female | HM | Prevention | Active | Not U. |
| Moyer-Gusé 2011 | Video | Mixed | RA | Prevention | Active | Not U. |
| Murphy 2015 | Video | Female | HM | Detection | Active | Not U. |
| Ochoa 2020 | Video | Female | HM | Detection | Active | Under-served |
| Peng 2009 | MM | Mixed | Nutrition | Prevention | Passive | Not U. |
| Saucier 2022 | Video | Female | RA | Prevention | Passive | Under-served |
| Solomon 1989 | Video | Mixed | RA | Prevention | Passive | Not U. |
| Underwood 2017 | Other | Mixed | Mixed | Prevention | Passive | Under-served |
| Unger 2013 | Writing | Female | GWB | Prevention | Active | Under-served |
| Yu 2010 | Writing | Mixed | RA | Prevention | Active | Not U. |

*Note*. MM = Multimodal. HM = Health maintenance. GWB = General well-being. RA = Risk avoidance. Not U. = Not Under-served.

Supplementary Table 3. General study data.

| Study | Number randomized | % Women | Age (in years) | Education: Highschool or more (%) |
| --- | --- | --- | --- | --- |
| Arendt 2018 | *N* = 273 | 74 | *M* = 26.93, *SD* = 10.08 | 89 |
| Baumeister 2021 | *N* = 219 | 77 | *M* = 24.60*, SD* = 5.86 | 100 |
| Beach 2020 | n.r. | 30 | *M* = 31.3, *SD* = 16.3 | n.r. |
| Betsch 2020* | *N* = 144 | 78.5 | *M* = 21.03*, SD* = 2.47 | 100 |
| Bekalu 2018 | *N* = 627 | 56 | 18-29: 20.7%, 30-44: 27.8%, 45-59: 29.8%, 60+: 21.7% | 84 |
| Bokhour 2016 | *N* = 618 | 8 | <50: 9.5%, 50-65: 52.4%, 65+: 38.1% | 93 |
| Cabassa 2015 | *N* =185 | 50 | *M* = 36.15, *SD* = 13.8 | 43 |
| Chang 2008 | *N* = 264 | 49 | n.r. | 100 |
| Clayton 2023 | *N* = 100 | 81 | *M* = 19.72, *SD* = 1.32 | 100 |
| Davis 2020** | *N* = 195 | 40 | *M* = 30.38, *SD* = 14.07 ^a^ | 10 |
| Davis 2021** | n.r. | n.r. | n.r. | n.r. |
| Feng 2021 | *N* = 365 | 51 | *M* = 37.1, *SD* = 11.8 | 69 |
| Forster 2016 | *N* = 334 | 39.5 | *M* = 53, *SD* = 12.53 | 53 |
| Gwede 2019 | *N* = 76 | 67 | *M* = 57.7, *SD* = 6.2 | 32 |
| Hernandez 2013 | *N* = 146 | 100 | 18-25: 7.0%, 26-35: 39.7%, 36-45: 36.1%, 46-55: 17.0% | 21 |
| Hoffman 2017 | *N* = 89 | 68 | *M* = 57.6, *SD* = 6.9 | At least college: 69% |
| Jiang 2021 | *N* = 527 | 51 | *M* = 34.78, *SD* = 14.19 | 100 |
| Jibaja 2000 | *N* = 178 | n.r. | n.r. | n.r. |
| Jones 2013 | *N* = 295 | n.r. | *M* = 22.05, *SD* = 3.5 | *M* = 12.18, *SD* = 1.5^b^ |
| Koops van’t Jagt 2013 | *N* = 202 | n.r. | n.r. | n.r. |
| Kreuter 2010 | *N* = 489 | 100 | *M* = 60.5, *SD* = 12.0 | 68 |
| Kreuter 2005 | *N* = 1227 | 100 | n.r. | n.r. |
| Lapinski 2008 | *N* = 100 | 37 | *M* = 27.62, *SD* = 5.71 | n.r. |
| Larkey 2015 | *N* = 539 | 66 | *M* = 58.4, *SD* = 6.5 | At least university: 22% |
| Limon 2004 | *N* = 141 | 66 | *M* = 20.3 | 100 |
| Liu 2020 | *N* = 439 | 61.5 | *M* = 20.34, *SD* = 2.34 | 100 |
| Liu 2021 | *N* = 303 | 100 | *M* = 30.41, *SD* = 6.15 | 99 |
| Liu 2023 | *N* = 252 | 73 | *M* = 33.93, *SD* = 11.84 | *M* = 15.91, *SD* = 4.32^c^ |
| Love 2009 | *N* = 489 | 100 | 18-35: 20.2%, 36-45: 20.2%, 46-55: 30.5%, 56-65: 17.1%, 66+: 12.0% | n.r. |
| McDonald 2006 | *N* = 113 | 100 | *M* = 42.6; *SD* = 12.41 | 87.5 |
| Moyer-Gusé 2011 | *N* = 437 | 78 | *M* = 19.8 | 100 |
| Murphy 2015 | *N* = 901 | 100 | 25-29: 11.2%, 30-34: 16.1%, 35-39: 26.1%, 40-45: 46.6% | 92 |
| Ochoa 2020 | *N* = 300 | 100 | n.r. | 50 |
| Peng 2009 | *N* = 40 | 80 | *M* = 20 | 100 |
| Saucier 2022** | n.r. | n.r. | *M* = 17.93, *SD* = 0.96 ^a^ | n.r. |
| Solomon 1989 | *N* = 182 | 20 | *Median* = 24 | 81 |
| Underwood 2017 | *N* =1200 | 50 | *M* = 34.85 | *M* = 4.3 ^c^ |
| Unger 2013 | *N* =185 | 47.5 | *M* = 35.8, *SD* = 12.9 | 37 |
| Yu 2010 | *N* =213 | n.r. | *M* = 19.98, *SD* = .87 | 100 |

*Note.* *Data extracted of Study 2.**Data extracted for the subgroup of participants ≥ 18 years. n.r.: not reported. ^a^: Data refer to the full age sample. ^b^: years in school. ^c^: years of education.

Supplementary Figure S1. Effects of Entertainment-Education on persuasion at post timepoint.


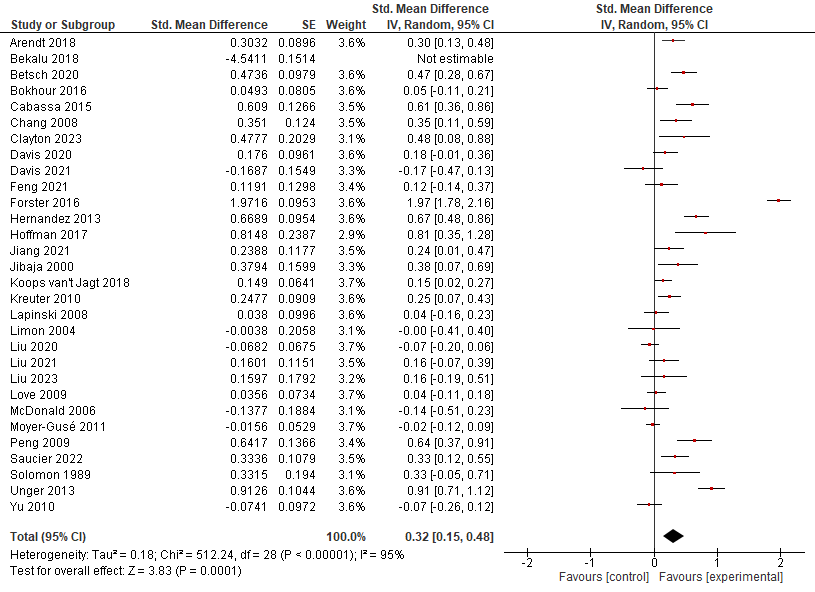


Supplementary Figure S2. Effects of Entertainment-Education on persuasion at follow-up timepoint.


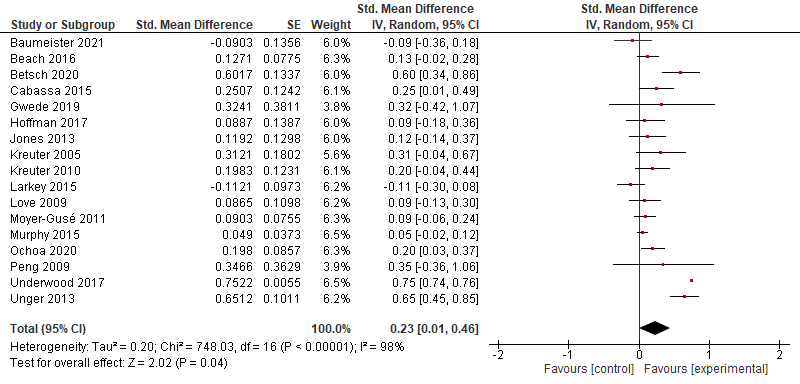


Supplementary Figure S3. Effect of the subgroup *type of control group.*


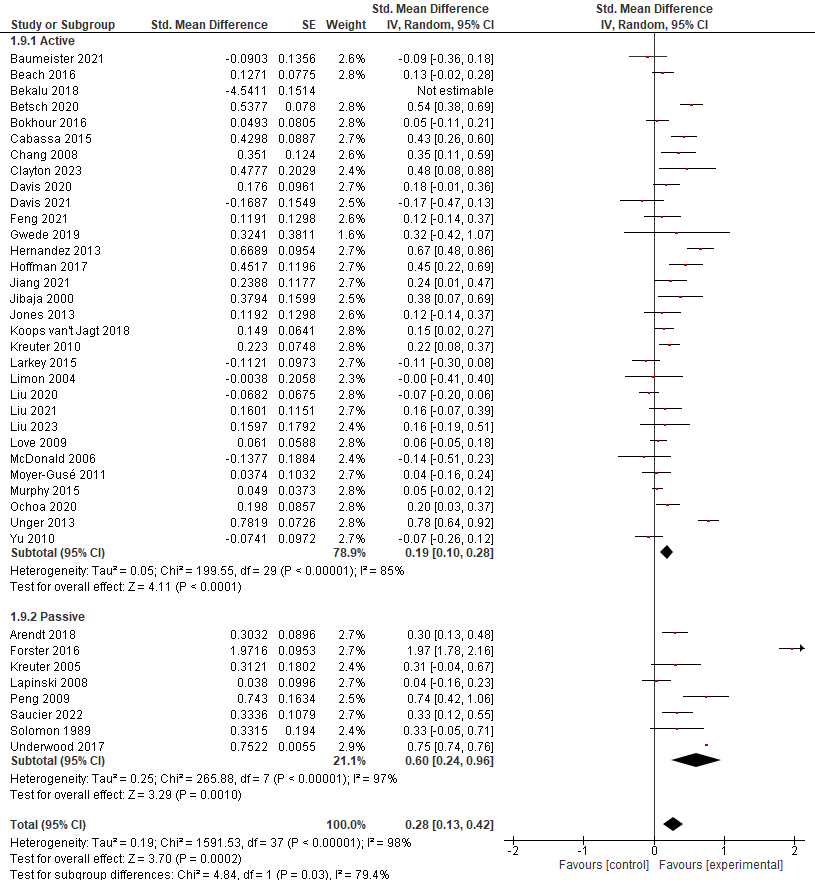


Supplementary Figure S4. Effect of the subgroup *taxonomy of health behavior.*


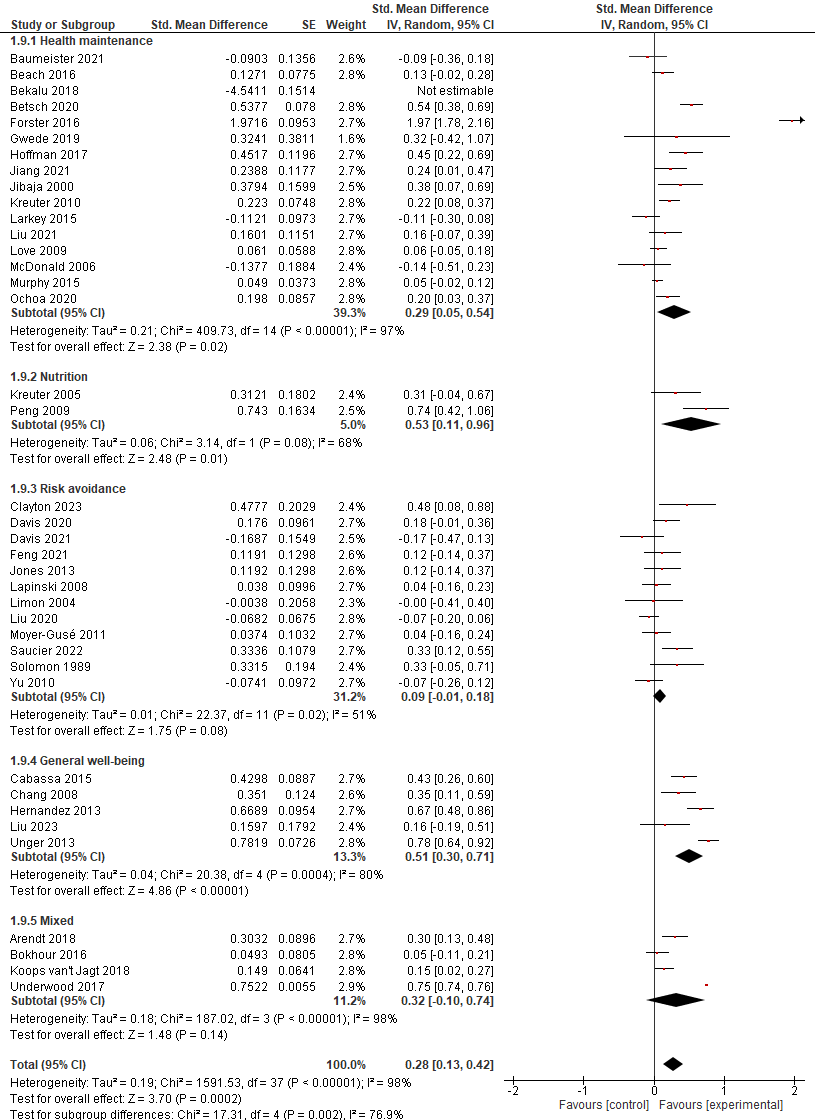


Supplementary Figure S5. Effect of the subgroup *type of health behavior.*


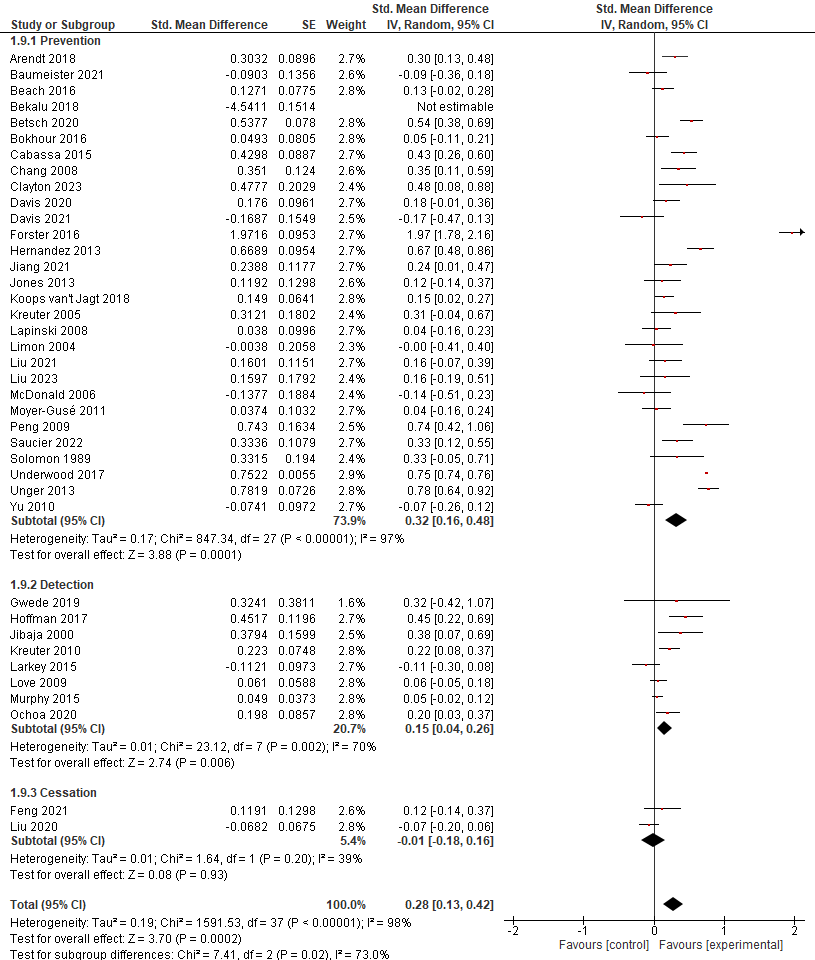


Supplementary Figure S6. Effect of the subgroup *delivery mode.*


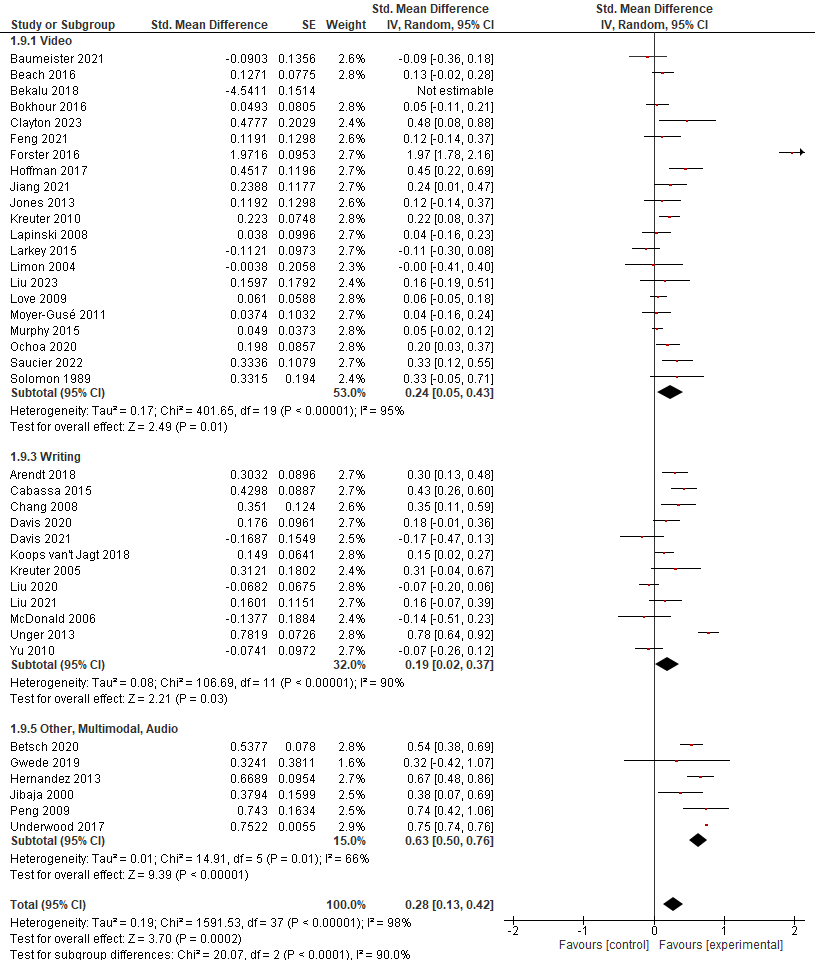


Supplementary Figure S7. Effect of the subgroup *gender.*


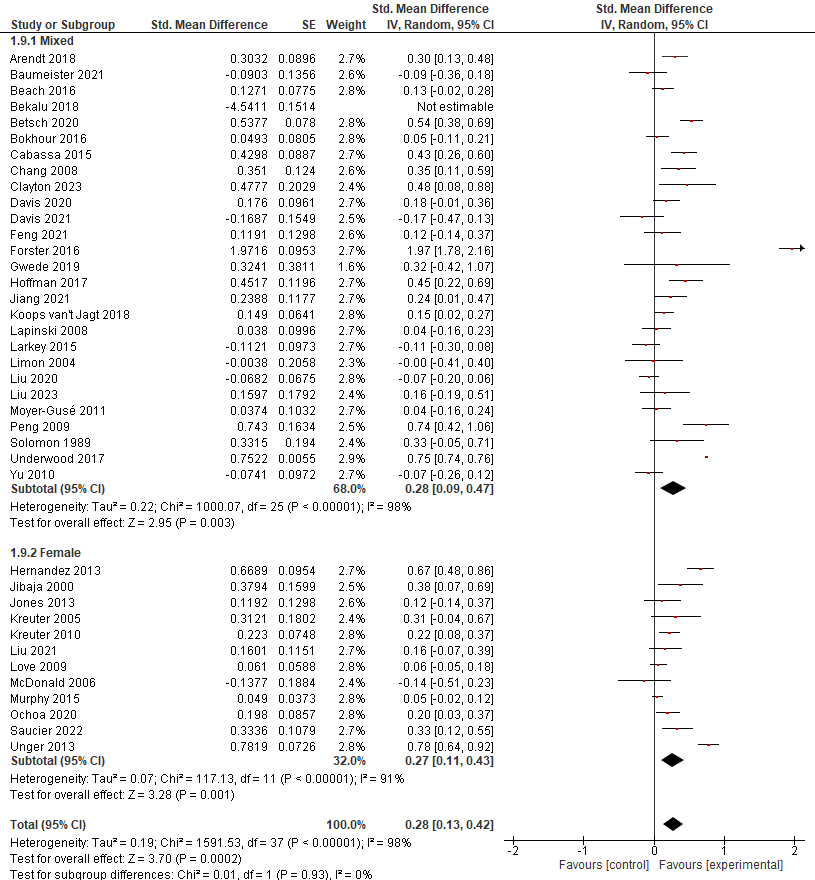


Supplementary Figure S8. Effect of the subgroup *healthcare.*


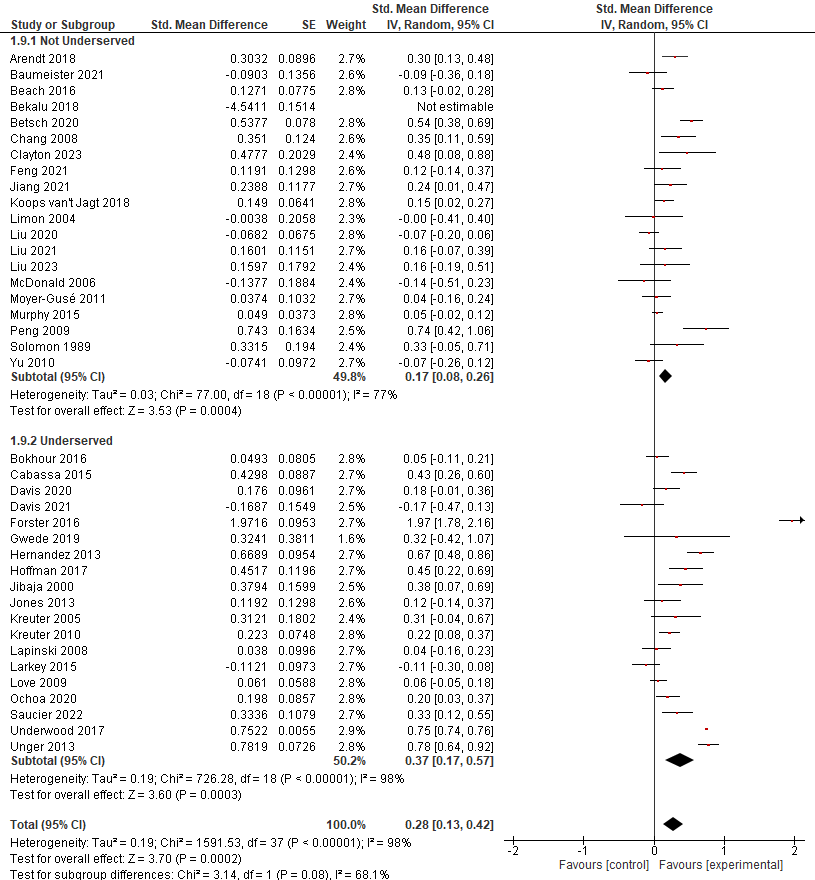


Supplementary Figure S9. Risk of bias graph for the nine assessed domains.


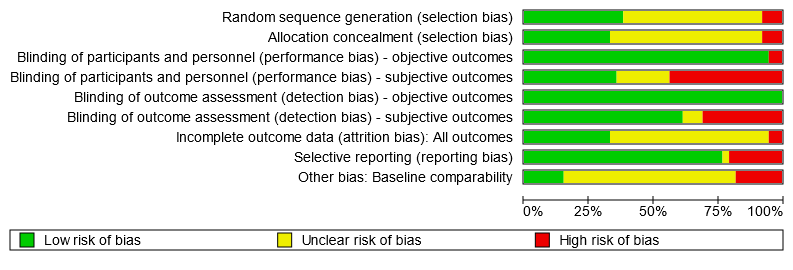


Supplementary Figure S10. Results of the Quality Assessment for the included studies.


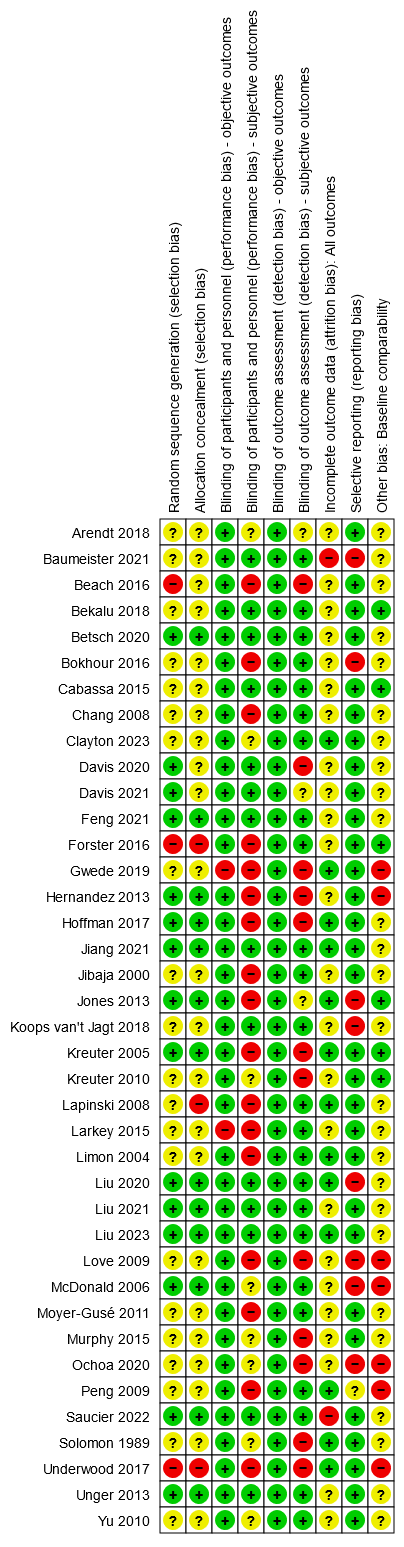


Supplementary Figure S11. Funnel plot.


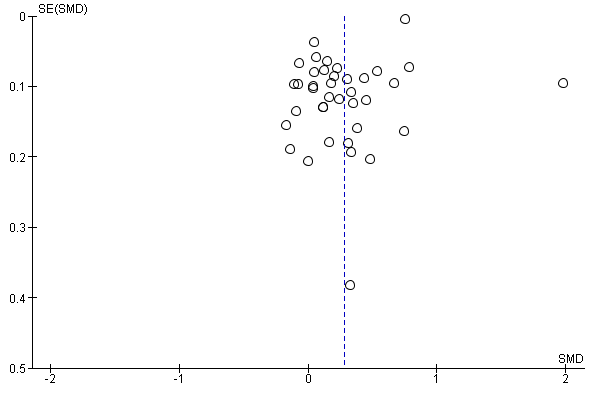


Appendix A. Pubmed Search strategy.

((health[mh] OR health communication[mh] OR persuasive communication [mh] OR health education[mh] OR prevention and control[mh] OR health promotion[mh]) AND (narration*[mh] OR entertainmenteducation[tiab] OR EE[tiab] OR drama*[tiab] OR edu-tainment[tiab] OR edutainment[tiab] OR entertainment[tiab] OR film*[tiab] OR mass media*[tiab] OR novela*[tiab] OR telenovela*[tiab] OR fotonovela*[tiab] OR comic*[tiab] OR radio[tiab] OR soap opera*[tiab] OR soap*[tiab] OR television[tiab] OR TV[tiab] OR serie*[tiab] OR show[tiab] OR theater*[tiab]) AND (Health Knowledge[mh] OR Attitude to health[mh] OR Health Knowledge, Attitudes, Practice[mh] OR knowledge[tiab] OR attitude*[tiab] OR behaviour*[tiab] OR behavior* [tiab] OR intention*[tiab] OR persuasion[tiab]) AND (randomized controlled trial[pt] OR controlled clinical trial[pt] OR randomized[tiab] OR placebo[tiab] OR drug therapy[sh] OR randomly[tiab] OR trial[tiab] OR controlled[tiab] OR groups[tiab] NOT (animals [mh] NOT humans [mh])))
